# Dielectric relaxation model of human blood as a superposition of Debye functions with relaxation times following a Modified-Weibull distribution

**DOI:** 10.1016/j.heliyon.2021.e06606

**Published:** 2021-03-26

**Authors:** Charandeep Singh Sodhi, Luan Carlos de Sena Monteiro Ozelim, Pushpa Narayan Rathie

**Affiliations:** a University of Texas at Dallas, Dallas, USA; b Department of Civil and Environmental Engineering, University of Brasilia, Brasília, Brazil; c Department of Statistics, University of Brasilia, Brasília, Brazil

**Keywords:** Gaussian distribution, Relaxation, Blood, Weibull distribution, Debye

## Abstract

Dielectric spectroscopy of the human blood is a powerful and convenient non-invasive testing technique that can be used to diagnose diseases such as diabetes and leukemia. One needs to consider rigorous experimental procedures and mathematical models to make the results of this type of test comparable. The present paper will discuss previously published results to further investigate the statistical modeling of the dielectric properties of human blood. The analysis shows that previously published results were related to Modified Weibull (MW) distributions of relaxation times, not Gaussian distributions, as reported. This re-analysis prevents the ill definition of fitting parameters, making sure they present physically justifiable values. Besides, for fluids presenting a Modified Weibull distribution of relaxation times, novel exact and closed-form expressions for the real and imaginary parts of complex permittivities were obtained in terms of generalized hypergeometric functions. Also, a high accuracy approximation was built for the imaginary part of the complex permittivity, creating an easy-to-use alternative expression for practitioners. The new results are used to fit experimental results for human blood, showing that more robust estimators are built for the parameters involved, which can be used as thresholds to classify the dielectric behavior of blood as normal (healthy) or anomalous (sick).

## 1 Introduction

As indicated by [1], the ability to anticipate and eventually intervene to avoid adverse future events is an extremely desirable goal to health professionals. Such predictions rely on the statistical modeling of the outcomes of tests, enabling health professionals to infer possible health issues by comparing the results of a test from a single individual to the possible results of tests performed on bigger number of similar individuals. Therefore, a rigorous statistical analysis allows the development of risk prediction techniques based on probabilities rather than intuition [1].

In this context, non-invasive testing techniques are quite advantageous, since they can be applied to a large number of individuals without the need of a complete medical structure (i.e. a hospital) to assist the tester and the subject being tested. Therefore, a large number of results can be recorded and used for future comparisons.

Besides being convenient and easy to perform, non-invasive methods are also commonly used these days for preventative healthcare as invasive methods are painful/inconvenient and can have a high reoccurring cost. Some of the drawbacks of invasive testing which are not observed in non-invasive methods are: invasive methods can lead to infection and invasive devices have stringent regulatory requirements.

When studying non-invasive tests to assess how the human body is affected by diseases, changes in blood's properties can be used to understand such impact. This comes from the fact that blood is a key body fluid, since it delivers oxygen to vital parts as well as transports nutrients, vitamins, and metabolites. Also, blood is a fundamental part of the immune system [2].

Researchers have to choose one of blood's properties to analyze and seek for aspects which characterize possible diseases. The study of the dielectric properties of biological systems and their components has shown to be important not only for fundamental scientific knowledge but also for applications in medicine, biology, biotechnology, and physiology [3]. Therefore, the dielectric behavior of blood can be chosen as the basis to propose non-invasive diagnostic techniques based on such biological fluid.

Regarding the dielectric properties of blood, pioneer studies at the beginning of last century allowed researchers to deduce from dielectric studies that erythrocytes are composed of a poorly conducting envelope enclosing a conducting electrolyte [4], [5].

From that time on, the dielectric properties of blood cells have been investigated. Recent studies report that non-invasive glucose monitoring in patients with diabetes can be performed by a system based on impedance spectroscopy [6]. Such system was attached to the skin of test subjects and the glucose monitoring was indirectly performed. In this case, the overall dielectric behavior is influenced not only by the blood, but also by the skin and other factors.

In order to further isolate the phenomenon of interest, the authors in [7] compared the dielectric properties of normal and diabetic blood collected from test individuals. Their study indicated that a proper modeling of the dielectric behavior of blood may lead to a powerful non-invasive technique to diagnose and control diabetic patients.

In a similar fashion, the author in [8] studied the low frequency dielectric properties of human blood, indicating that their results suggest new diagnostic and therapeutic methods for blood disorders.

This is also the case of a recently introduced non-invasive method of detecting leukemia [9], where red blood cells are subjected to the application of an alternating current and the electrical resistance is measured. Such methodology indicates the electrical resistance will show a peak at a characteristic resonant frequency, which is different for cancerous blood cells than for normal blood cells.

Literature also reveals that the dielectric parameters of blood are relevant for various other medical applications like cell separation (e.g., cancer cells from normal blood cells), checking the deterioration of preserved blood and dielectric coagulometry [2].

In order to properly use dielectric measurements to diagnose diseases, the physical and mathematical modeling of this phenomenon must be carried out with extreme care. Therefore, studies on this subject are of utmost importance.

This way, to better understand how blood behaves when subjected to an electrical field, the effects of the blood-microstructure on its electrical conductivity have been reported in the literature [7]. The authors in [10] also studied the role of erythrocytes and leucocytes in charge transfer through human blood.

Literature indicates that experimental dielectric relaxation data for human blood show a deviation from the classical Debye model [11]. This deviation may be attributed to the fact that blood does not have a single relaxation time, but actually it presents a certain distribution of relaxation times. Authors in [11] carried out a study to understand if the dielectric modeling of relaxation through human blood could benefit from considering multiple relaxation times which were distributed as a Gaussian distribution.

It is clear that human blood is a complex fluid whose electrical interaction with external fields is intricate. On the other hand, the studies cited have provided valuable insights about the modeling of such interaction.

Even though the reported papers covered some difficult modeling issues, it was observed that some mathematical results needed further clarification. In special, some of the mathematical results presented in [11] can be re-derived to better accommodate some statistical concepts which were considered by such authors.

Therefore, in the present paper, the physiological understanding of how dielectric properties are affected by diseases is not of central interest as we only focused on the mathematical modeling of such changes. Specially, we point out some issues in previously published results as well as discuss some of the statistical features of the models explored in the literature. By doing so, we hope to enhance the mathematical models used to fit experimental results, leading to more robust feature estimations and, ultimately, a better definition of the parameters' thresholds to classify the dielectric behavior as normal (healthy) or anomalous (sick).

In Section 2 we shall revisit the basic formulation behind dielectric relaxation models. Section 3, on the other hand, presents the definition of some random variables of interest. Besides, Section 4 discusses previously published results and presents the re-derived equations. Section 5 presents novel exact and approximate expressions for real and complex permittivities when the distribution of relaxation times follow a Modified-Weibull distribution. We then present a practical application of the new equations, by using them to fit experimental data in Section 6. Also, in Section 7, we discuss how the identifiability of Modified-Weibull random variables may be an issue while using the equations reported in the literature and hereby developed. Conclusions are then presented in Section 8. In order to better familiarize the reader, the Appendix presents some of the special functions which will be used in the present paper, as well as their mathematical definitions. Besides, some other relevant mathematical definitions are also presented.

## 2 Revisiting relaxation models

As previously indicated, experimental dielectric relaxation data for human blood show a deviation from the classical Debye model due to the fact that blood does not have a single relaxation time, but actually it presents a certain distribution of relaxation times [11]. This can be thought of as the superposition of parallel combinations of linear series RC circuits with different time constants [12].

This way, to account for this distribution, the complex permittivity of a species modeled by a superposition of Debye functions with relaxation times following a statistical distribution with probability density function (pdf) f(τ) is [12]:

(1) $$\varepsilon^{⁎}=\varepsilon_{\infty }+(\varepsilon_{DC}-\varepsilon_{\infty })\int_{0}^{\infty }\frac{f(\tau )}{1+jw\tau }d\tau -\frac{j\sigma_{DC}}{w\varepsilon_{0}}$$

where $j=\sqrt{-1}$, $\varepsilon^{⁎}$ is the complex permittivity at frequency w/(2π), $\varepsilon_{\infty }$ is the dielectric constant at very high values of the frequency (the real part of the complex $\varepsilon^{⁎}$), $\varepsilon_{DC}$ is the dielectric constant under DC conditions (zero frequency), *τ* is the relaxation time of the dipoles (where each micro-particle represents an electric dipole), $\sigma_{DC}$ is the DC ionic conductivity and $\varepsilon_{0}$ is the permittivity of free space. It is easy to prove that the real ($\varepsilon^{\prime }$) and imaginary ($\varepsilon^{\prime \prime }$) parts of the complex permittivity are given as $\varepsilon^{⁎}=\varepsilon^{\prime }-\varepsilon^{\prime \prime }j$, where:

(2) $$\varepsilon^{\prime }=\varepsilon_{\infty }+(\varepsilon_{DC}-\varepsilon_{\infty })\int_{0}^{\infty }\frac{f(\tau )}{1+w^{2}\tau^{2}}d\tau$$

(3) $$\varepsilon^{\prime \prime }=(\varepsilon_{DC}-\varepsilon_{\infty })\int_{0}^{\infty }\frac{w\tau f(\tau )}{1+w^{2}\tau^{2}}d\tau +\frac{\sigma_{DC}}{w\varepsilon_{0}}$$

where:

(4) $$\int_{0}^{\infty }f(\tau )d\tau =1$$

As previously indicated, let *T* be the random variable (RV) which represents the statistical distribution of relaxation times. Then, f(τ) can be interpreted as its pdf.

One shall notice that (4) indicates that RV *T* is a positive RV, i.e., only takes positive values. This is obvious as *T* represents the distribution of relaxation times, which are positive real numbers.

Also, if *T* has a pdf given as f(τ), then, by the Jacobian rule [13], the random variable L=ln⁡T has pdf g(l) given as $g(l)=f(e^{l})e^{l}$ or g(ln⁡τ)=f(τ)τ. This explains, in statistical terms, why it is possible to investigate the logarithm of the relaxation times, instead of the relaxation times themselves. This leads to the following relation [14]:

(5) $$\int_{-\infty }^{\infty }g(l)dl=\int_{-\infty }^{\infty }g(\ln\tau )d\ln\tau =1$$

A common source of confusion between researchers is to directly link (4) and (5) without paying attention to the fact that in order to maintain consistency, g(ln⁡τ)=τf(τ) [14]. Therefore, if one wants to model the distribution of relaxation times, the support of the RV *T* is the positive real axis, while the support of the RV L=ln⁡T becomes the whole real axis.

It is important to highlight that considering the distribution of relaxation times as a continuous RV is a simplification. The discrete nature of blood-microparticles may affect this assumption. On the other hand, for practical purposes, literature results such as the ones in [2] indicate this assumption reasonably holds. Besides, to avoid some practical drawbacks related to considering relaxation times ranging from 0 to ∞, one must select distributions with virtually null cumulative probabilities on their tails.

In order to present our main results, we should introduce some RVs of interest.

## 3 Random variables of interest

Let *X* be a RV assuming real values, x∈R, whose pdf is:

(6) $$f(x,\mu ,\sigma )=\frac{1}{\sigma }\phi \left(\frac{x-\mu }{\sigma }\right)$$

where μ∈R and $\sigma^{2}>0$ are the distributions parameters representing its mean and variance, respectively, and:

(7) $$\phi (\xi )=\frac{1}{\sqrt{2\pi }}\exp\left(-\frac{1}{2}\xi^{2}\right)$$

We say *X* is distributed as a Normal Distribution, N(μ,σ), and write X∼N(μ,σ). Besides, let *Z* be a RV assuming positive values, z≥γ, whose pdf is:

(8) $$h(z,\beta ,\eta ,\gamma )=\frac{\beta }{\eta }{\left(\frac{z-\gamma }{\eta }\right)}^{\beta -1}\exp\left(-{\left(\frac{z-\gamma }{\eta }\right)}^{\beta }\right)$$

where γ∈R, β>0 and η>0 are the distributions parameters. We say *Z* follows a 3-parameter Weibull distribution and write Z∼W(β,η,γ). A particular case, when β=2 and γ=0, of such RV can be further studied. Thus, the pdf of $Z_{\left(2,0\right)}∼W(2,\eta ,0)$, where z≥0, is:

(9) $$h_{\left(2,0\right)}(z,\eta )=\frac{2}{\eta }\left(\frac{z}{\eta }\right)\exp\left(-{\left(\frac{z}{\eta }\right)}^{2}\right)$$

It is not possible to account for a location parameter *γ* inside and outside of the exponential function in (9) and also get the support of $Z_{\left(2,0\right)}$ to be z≥0. One can, on the other hand, include the location parameter *γ* only inside the exponential function and then normalize (9) by a multiplicative constant *c* which makes sure that the integral over the real half-line is equal to 1. Thus, we can define a new Modified-Weibull RV *U*, with support u≥0 and whose pdf is:

(10) $$q(u,\eta ,\gamma )=\frac{2}{\eta c}\left(\frac{u}{\eta }\right)\exp\left(-{\left(\frac{u-\gamma }{\eta }\right)}^{2}\right)$$

where γ∈R and η>0 are the distributions parameters. We say U∼MW(η,γ). The constant *c* can be obtained by noticing that:

(11) $$\int_{0}^{\infty }q(u,\eta ,\gamma )du=1\Rightarrow \int_{0}^{\infty }\frac{2}{\eta }\left(\frac{u}{\eta }\right)\exp\left(-{\left(\frac{u-\gamma }{\eta }\right)}^{2}\right)du=c$$

This way, by using integration by parts, it is easy to prove that:

(12) $$c=e^{-\frac{\gamma^{2}}{\eta^{2}}}+\frac{\sqrt{\pi }\gamma }{\eta }\left(1+\text{erf}\left(\frac{\gamma }{\eta }\right)\right)$$

Now that we have presented the random variables which will be used in the present paper, we may proceed to the analysis of previously published results.

## 4 Mathematical results of [11]

In [11], the author studied the dielectrical relaxation of human blood. He indicated that blood is constituted by red blood cells (RBCs), white blood cells (WBCs), micro-light blood particles (MLPs, which are lighter than RBCs and WBCs) and other microparticles. Also, he indicates that dielectric measurements of blood are influenced by all these cells/particles.

At first, the author presents the so-called Cole equation, where the parameter *α* is an exponent of the imaginary quantity *jwτ*. The Cole equation is described as:

(13) $$\varepsilon^{⁎}=\varepsilon_{\infty }+\frac{\left(\varepsilon_{DC}-\varepsilon_{\infty }\right)}{1+{\left(jw\tau \right)}^{\alpha }}$$

where *α* is a parameter ranging between 0 and 1 with dimensionless units. The author in [11] indicates that *α* is the so-called Cole parameter, but that is not accurate. Actually, the Cole parameter, $\alpha_{c}$, satisfies $\alpha_{c}=1-\alpha$ in (13).

Besides, following the nomenclature on [11], let *n* be the number of dipoles of one type per unit volume; then one can write for the three types of cells/particles in blood:

(14) $$n={\left(n_{0}\right)}_{WBC}f{\left(\tau \right)}_{WBC}+{\left(n_{0}\right)}_{RBC}f{\left(\tau \right)}_{RBC}+{\left(n_{0}\right)}_{MLP}f{\left(\tau \right)}_{MLP}$$

where $f{\left(\tau \right)}_{k}$ is the distribution function of the relaxation times for each particle *k*. One should notice that (14) is not correct, as it should be in a differential form since we are dealing with probability density functions. Thus, the correct writing would be:

(15) $$dn=\left({\left(n_{0}\right)}_{WBC}f{\left(\tau \right)}_{WBC}+{\left(n_{0}\right)}_{RBC}f{\left(\tau \right)}_{RBC}+{\left(n_{0}\right)}_{MLP}f{\left(\tau \right)}_{MLP}\right)d\tau$$

We shall highlight that in (15) we are just weighting the distribution of relaxation times by the number of each respective blood-microparticle. Factors like mass, volume, elasticity and shape are accounted for by the distribution of relaxation times. Therefore, it is understood that, macroscopically, the distribution of relaxation times for each blood micro-particle is a random variable and that is what is modeled.

After that, in [11], the author indicates that:

(16) $$\int_{0}^{\infty }\left({\left(n_{0}\right)}_{WBC}f{\left(\tau \right)}_{WBC}+{\left(n_{0}\right)}_{RBC}f{\left(\tau \right)}_{RBC}+{\left(n_{0}\right)}_{MLP}f{\left(\tau \right)}_{MLP}\right)d\tau =1$$

Again, the results in [11] are not correct, as the integral would be equal to the total number of dipoles *n*, instead. The expressions in [11] would only be correct if ${\left(n_{0}\right)}_{k}$ represented the relative number of each cell/particle *k* with respect to the total number of dipoles. In that case, ${\left(n_{0}\right)}_{k}\leq 1$. To represent this normalized version of the number of cells/particles, we can use the values $r_{WBC}$, $r_{RBC}$ and $r_{MLP}$ for WBCs, RBC, and MLPs, respectively. Thus, a correct version of (16) would be:

(17) $$\int_{0}^{\infty }\left(r_{WBC}f{\left(\tau \right)}_{WBC}+r_{RBC}f{\left(\tau \right)}_{RBC}+r_{MLP}f{\left(\tau \right)}_{MLP}\right)d\tau =1$$

Equation (17) reveals that when multiple cells/particles are considered, the distribution of relaxation times of blood is a statistical mixture of the distributions of each cell/particle. This is important to highlight, as the statistical treatment of mixtures have particular issues to be considered. The identifiability of the mixtures of Modified Weibull Distributions will be later discussed in the present paper.

Thus, we can define the pdf of the distribution of relaxation times in blood as $f{\left(\tau \right)}_{blood}$ and write:

(18) $$f{\left(\tau \right)}_{blood}=r_{WBC}f{\left(\tau \right)}_{WBC}+r_{RBC}f{\left(\tau \right)}_{RBC}+r_{MLP}f{\left(\tau \right)}_{MLP}$$

The author of [11] indicates that, since RBCs constitute 99% of blood, only those cells were considered. This way, he states that:

(19) $$\int_{0}^{\infty }{\left(n_{0}\right)}_{RBC}f{\left(\tau \right)}_{RBC}d\tau =1$$

Once more, (19) is not correct. The consistent equations would be:

(20) $$\int_{0}^{\infty }{\left(n_{0}\right)}_{RBC}f{\left(\tau \right)}_{RBC}d\tau \approx n$$

and

(21) $$\int_{0}^{\infty }f{\left(\tau \right)}_{RBC}d\tau =1$$

as, regardless of each blood cell/particle, (21) is always valid.

Also in [11], the author introduced f(τ) and stated that it followed a Gaussian Distribution (GD) with pdf:

(22) $$f(\tau )=\frac{\tau }{T_{s}\sqrt{2\pi }}\exp\left(-\frac{{\left(\tau -\tau_{c}\right)}^{2}}{2T_{s}^{2}}\right),\;\tau \geq 0$$

A first important remark we must make is that RVs representing the distribution of relaxation times cannot follow a Normal distribution, as the latter can also take negative values. This issue indicates the nomenclature used by [11] is misleading.

Besides, as (4) and (21) indicate, f(τ) is a probability density function, thus it must integrate to 1 over its support. By integrating (22) from 0 to ∞, by using (11) and (12) with $\gamma =\tau_{c}$ and $\eta =\sqrt{2}T_{s}$, one may see that:

(23) $$\int_{0}^{\infty }\frac{\tau }{T_{s}\sqrt{2\pi }}\exp\left(-\frac{{\left(\tau -\tau_{c}\right)}^{2}}{2T_{s}^{2}}\right)d\tau =\frac{T_{s}}{\sqrt{2\pi }}\left(e^{-\frac{\tau_{c}^{2}}{2T_{s}^{2}}}+\frac{\sqrt{\pi }\tau_{c}}{\sqrt{2}T_{s}}\left(1+\text{erf}\left(\frac{\tau_{c}}{\sqrt{2}T_{s}}\right)\right)\right)$$

Equation (23) indicates that the results presented in [11] are not consistent with the theoretical constraints on f(τ).

Also, by looking at the definition of the pdf of a Gaussian RV in (6) and (7), a careful analysis reveals that there is no linear term *x* multiplying the exponential function in such definition. On the other hand, (22) and (23) show there is a *τ* term pre-multiplying the exponential function. Therefore, (22) does not represent the pdf of a Gaussian RV. This is the core of the issues we are addressing in the present paper.

Besides, visual inspection reveals that (22) resembles (9), except for the location parameter inside the exponential function. Actually, the RV described in [11], if properly normalized by the constant on the right-hand side of (23), follows a Modified-Weibull distribution with parameters $\gamma =\tau_{c}$ and $\eta =\sqrt{2}T_{s}$, whose pdf is given in (10).

Even though the author in [11] indicates the paper is about RBCs, he presents the results for all blood cells without considering the weights $r_{k}$, for each cell/particle *k*. In order to account for all the cells/particles, the mixture of distributions in (18) must be considered.

Mathematically, by using (2), (3) and (18), let the sub-index *k* refer to each of the blood cells/particles, such that 1, 2 and 3 refer to WBCs, RBCs and MLPs, respectively. Then:

(24) $$\varepsilon^{\prime }=\varepsilon_{\infty }+(\varepsilon_{DC}-\varepsilon_{\infty })\sum_{k=1}^{3}r_{k}\int_{0}^{\infty }\frac{f{\left(\tau \right)}_{k}}{1+w^{2}\tau^{2}}d\tau$$

(25) $$\varepsilon^{\prime \prime }=(\varepsilon_{DC}-\varepsilon_{\infty })\sum_{k=1}^{3}r_{k}\int_{0}^{\infty }\frac{w\tau f{\left(\tau \right)}_{k}}{1+w^{2}\tau^{2}}d\tau +\frac{\sigma_{DC}}{w\varepsilon_{0}}$$

where $r_{k}={\left(n_{0}\right)}_{k}/n$, k=1,2,3 and $\sum r_{k}=1$, according to the nomenclature of [11].

By considering the correct version of the pdfs involved as given in (10) with parameters $\gamma_{k}=\tau_{c,k}$ and $\eta_{k}=\sqrt{2}T_{s,k}$, (24) and (25) become:

(26) $$\varepsilon^{\prime }=\varepsilon_{\infty }+(\varepsilon_{DC}-\varepsilon_{\infty })\sum_{k=1}^{3}r_{k}\int_{0}^{\infty }\frac{\tau }{T_{s,k}^{2}(1+w^{2}\tau^{2})c_{k}}\exp\left(-\frac{{\left(\tau -\tau_{c,k}\right)}^{2}}{2T_{s,k}^{2}}\right)d\tau$$

(27) $$\varepsilon^{\prime \prime }=(\varepsilon_{DC}-\varepsilon_{\infty })\sum_{k=1}^{3}r_{k}\int_{0}^{\infty }\frac{w\tau^{2}}{T_{s,k}^{2}(1+w^{2}\tau^{2})c_{k}}\exp\left(-\frac{{\left(\tau -\tau_{c,k}\right)}^{2}}{2T_{s,k}^{2}}\right)d\tau +\frac{\sigma_{DC}}{w\varepsilon_{0}}$$

where:

(28) $$c_{k}=e^{-\frac{\tau_{c,k}^{2}}{2T_{s,k}^{2}}}+\frac{\sqrt{\pi }\tau_{c,k}}{\sqrt{2}T_{s,k}}\left(1+\text{erf}\left(\frac{\tau_{c,k}}{\sqrt{2}T_{s,k}}\right)\right)$$

Equations (26) and (27) are the correct versions of equations (9) and (8) of [11], respectively. Similarly, equations (11) to (13) of [11] need to be corrected to account for the relative number of each cell/particle $r_{k}$.

One of the main issues related to the results presented by [11] is that his results did not incorporate the effect of the normalizing constant $c_{k}$. Thus, when performing the fitting procedures, the values of $r_{k}$ and $\left(\varepsilon_{DC}-\varepsilon_{\infty }\right)$ may become unreasonable as $c_{k}$ gets out of the integral and impacts those parameters. This shall be illustrated in Section 6, where experimental data are fitted using the equations previously discussed.

Now that we have presented the re-analyzed versions of the equations deduced in [11], we shall introduce new exact and approximate expressions for the integrals in (26) and (27).

## 5 Exact and approximate expressions for permittivities

Even in [11], the integrals in (26) and (27) were not obtained in a closed form. In the present section, both exact and approximate expressions are obtained for such equations.

### 5.1 Exact expressions

In order to obtain the exact expressions, let us consider the variable change wτ=y on both (26) and (27). This leads to:

(29) $$\varepsilon^{\prime }=\varepsilon_{\infty }+(\varepsilon_{DC}-\varepsilon_{\infty })\sum_{k=1}^{3}\frac{r_{k}}{w^{2}T_{s,k}^{2}c_{k}}\int_{0}^{\infty }\frac{y}{1+y^{2}}\exp\left(-\frac{{\left(y-w\tau_{c,k}\right)}^{2}}{2w^{2}T_{s,k}^{2}}\right)dy$$

(30) $$\varepsilon^{\prime \prime }=(\varepsilon_{DC}-\varepsilon_{\infty })\sum_{k=1}^{3}\frac{r_{k}}{w^{2}T_{s,k}^{2}c_{k}}\int_{0}^{\infty }\frac{y^{2}}{1+y^{2}}\exp\left(-\frac{{\left(y-w\tau_{c,k}\right)}^{2}}{2w^{2}T_{s,k}^{2}}\right)dy+\frac{\sigma_{DC}}{w\varepsilon_{0}}$$

Therefore, it is easy to see that both permittivity values depend on an integral $I_{p}(a,b)$ of the type:

(31) $$I_{p}(a,b)=\int_{0}^{\infty }\frac{y^{p}}{1+y^{2}}\exp\left(-{\left(\frac{y-a}{b}\right)}^{2}\right)dy$$

The exact expressions can be obtained both in a closed-form, in terms of generalized hypergeometric functions, as well as an infinite series.

#### 5.1.1 Closed-form expressions

Literature [13] reveals that:

(32) $${\left(1+x\right)}^{-a}=\frac{1}{\Gamma (a)}H_{1,1}^{1,1}\left[x\;|\begin{array}{c} \left(1-a,1\right)\\ \left(0,1\right) \end{array}\right]=\frac{1}{2\pi j\Gamma (a)}\int_{L^{⁎}}\Gamma (s)\Gamma (a-s)x^{-s}ds$$

where $j=\sqrt{-1}$ and $L^{⁎}$ is a suitable contour separating the poles of Γ(s) from Γ(a−s).

This way, from (32) and (31):

(33) $$I_{p}(a,b)=\frac{1}{2\pi j}\int_{L^{⁎}}\Gamma (s)\Gamma (1-s)\int_{0}^{\infty }y^{p-s}\exp\left(-{\left(\frac{y-a}{b}\right)}^{2}\right)dyds$$

Now we may introduce a main result which shall be used throughout the paper.

Theorem 1 *Whenever* $ℜ(\alpha_{1})>-1$*, the following integral can be expressed in a closed form in terms of Generalized Hypergeometric functions:*

(34) $$\int_{0}^{\infty }y^{\alpha_{1}}\exp\left(-{\left(\frac{y-\alpha_{2}}{\alpha_{3}}\right)}^{2}\right)dy={\left(-\alpha_{2}\right)}^{\alpha_{1}+1}\Gamma (\alpha_{1}+1)H_{1,2}^{2,0}\left[\frac{\alpha_{2}^{2}}{\alpha_{3}^{2}}\;|\begin{array}{c} \left(0,2\right)\\ \left(0,1),(-\alpha_{1}-1,2\right) \end{array}\right]={\left(-\frac{\alpha_{2}}{2}\right)}^{\alpha_{1}+1}\Gamma (\alpha_{1}+1)G_{1,2}^{2,0}\left[\frac{\alpha_{2}^{2}}{\alpha_{3}^{2}}\;|\begin{array}{c} 1/2\\ -\frac{\alpha_{1}+1}{2},-\frac{\alpha_{1}}{2} \end{array}\right]$$

Proof At first, one shall consider the left hand side of (34) and consider the contour integral representation of the exponential function, given as [13]:

(35) $$\exp(-z)=\frac{1}{2\pi j}\int_{L^{⁎}}\Gamma (s)z^{-s}ds$$

Thus, by combining (34) and (35), one gets:

(36) $$\int_{0}^{\infty }y^{\alpha_{1}}\exp\left(-{\left(\frac{y-\alpha_{2}}{\alpha_{3}}\right)}^{2}\right)dy=\frac{1}{2\pi j}\int_{L^{⁎}}\Gamma (s)\int_{0}^{\infty }y^{\alpha_{1}}{\left(\frac{y-\alpha_{2}}{\alpha_{3}}\right)}^{-2s}dyds$$

By performing the variable change $y=\alpha_{2}x/(x-1)$, the right hand side of (36) becomes:

(37) $$\frac{1}{2\pi j}\int_{L^{⁎}}\Gamma (s)\int_{0}^{\infty }y^{\alpha_{1}}{\left(\frac{y-\alpha_{2}}{\alpha_{3}}\right)}^{-2s}dyds=\frac{{\left(-\alpha_{2}\right)}^{\alpha_{1}+1}}{2\pi j}\int_{L^{⁎}}\Gamma (s){\left(-\frac{\alpha_{2}}{\alpha_{3}}\right)}^{-2s}\int_{0}^{1}x^{\alpha_{1}}{\left(1-x\right)}^{2s-\alpha_{1}-2}dxds$$

The Beta function B(x,y) can be defined as [13]:

(38) $$B(z_{1},z_{2})=\int_{0}^{1}q^{z_{1}-1}{\left(1-q\right)}^{z_{2}-1}dq=\frac{\Gamma (z_{1})\Gamma (z_{2})}{\Gamma (z_{1}+z_{2})}$$

for $ℜ(z_{1})>0$ and $ℜ(z_{2})>0$.

This way, (37) can be represented in terms of the Beta function by using (38), leading to:

(39) $$\frac{1}{2\pi j}\int_{L^{⁎}}\Gamma (s)\int_{0}^{\infty }y^{\alpha_{1}}{\left(\frac{y-\alpha_{2}}{\alpha_{3}}\right)}^{-2s}dyds=\frac{{\left(-\alpha_{2}\right)}^{\alpha_{1}+1}}{2\pi j}\int_{L^{⁎}}\Gamma (s){\left(-\frac{\alpha_{2}}{\alpha_{3}}\right)}^{-2s}\frac{\Gamma (\alpha_{1}+1)\Gamma (2s-\alpha_{1}-1)}{\Gamma (2s)}ds$$

whenever $ℜ(\alpha_{1})>-1$ and $ℜ(s)>(\alpha_{1}+1)/2$.

One may see that, by using the contour integral representation of the H-function given in (72), the first representation of the integral in (34) has been proven. On the other hand, to get the Meijer-G function representation, one may notice that the Gamma function multiplication theorem indicates that [13]:

(40) $$\prod_{j=0}^{k-1}\Gamma \left(z+\frac{j}{k}\right)=\Gamma (kz){\left(2\pi \right)}^{\frac{k-1}{2}}k^{\frac{1}{2}-kz}$$

Therefore, by directly using (40) with k=2, (39) becomes:

(41) $$\frac{1}{2\pi j}\int_{L^{⁎}}\Gamma (s)\int_{0}^{\infty }y^{\alpha_{1}}{\left(\frac{y-\alpha_{2}}{\alpha_{3}}\right)}^{-2s}dyds={\left(-\frac{\alpha_{2}}{2}\right)}^{\alpha_{1}+1}\frac{\Gamma (\alpha_{1}+1)}{2\pi j}\int_{L^{⁎}}\frac{\Gamma \left(s-\frac{\alpha_{1}+1}{2}\right)\Gamma \left(s-\frac{\alpha_{1}}{2}\right)}{\Gamma \left(s+\frac{1}{2}\right)}{\left(-\frac{\alpha_{2}}{\alpha_{3}}\right)}^{-2s}ds$$

Now, by using the contour integral representation of the H-function given in (72) and noticing that, by definition, the Meijer-G function is the H-function with $A_{j},B_{j}=1$, ∀*j*, the second representation of the integral in (34) has been proven, which ends the proof. □

This way, by means of Theorem 1, (33) can be rewritten as:

(42) $$I_{p}(a,b)=\frac{1}{2\pi j}\int_{L^{⁎}}\Gamma (s)\Gamma (1-s){\left(-a\right)}^{p-s+1}\Gamma (p-s+1)H_{1,2}^{2,0}\left[\frac{a^{2}}{b^{2}}\;|\begin{array}{c} \left(0,2\right)\\ \left(0,1),(s-p-1,2\right) \end{array}\right]ds=\frac{1}{2\pi j}\int_{L^{⁎}}\Gamma (s)\Gamma (1-s){\left(-\frac{a}{2}\right)}^{p-s+1}\Gamma (p-s+1)G_{1,2}^{2,0}\left[\frac{a^{2}}{b^{2}}\;|\begin{array}{c} 1/2\\ -\frac{p-s+1}{2},-\frac{p-s}{2} \end{array}\right]ds$$

which is valid when ℜ(s)<p+1.

The results in (42), indicate that $I_{p}(a,b)$ may be represented as a H-function of two variables or, even simpler, as a Meijer-G function of two variables.

On the other hand, we can use another trick to obtain a series representation for $I_{p}(a,b)$ which converges nicely and fast.

#### 5.1.2 Infinite series

We will use an approach similar to [15] to build exponential approximations to power functions. Thus, let one consider the following integral:

(43) $$x^{-1}=\int_{0}^{\infty }\exp(-tx)dt$$

Thus, we can use (43) and write:

(44) $${\left(1+y^{2}\right)}^{-1}=\int_{0}^{\infty }\exp(-t)\exp(-ty^{2})dt$$

It is widely known that integrals as the one presented on the right hand side of (44) can be easily calculated by means of a Gauss-Laguerre quadrature, which states that [16]:

(45) $$\int_{0}^{\infty }\exp(-t)h(t)dt\approx \sum_{i=1}^{n}q_{i}h(x_{i}),$$

where the values $x_{i}$ are the *i*-th root of Laguerre polynomial $L_{n}(x)$ and the weights $q_{i}$ are given as:

(46) $$q_{i}=\frac{x_{i}}{{\left(n+1\right)}^{2}{\left[L_{n+1}\left(x_{i}\right)\right]}^{2}}.$$

In order to make the approximation in (45) an equality, it suffices to take *n* as big to ensure the accuracy required. Thus, from (31) and (45), $I_{p}(a,b)$ can be given as:

(47) $$I_{p}(a,b)=\lim_{n\to \infty }\sum_{i=1}^{n}q_{i}\int_{0}^{\infty }y^{p}\exp\left(-{\left(\frac{y-a}{b}\right)}^{2}-x_{i}y^{2}\right)dy$$

By rearranging the terms inside the integral in (47) and using Theorem 1, the following result holds:

(48) $$\int_{0}^{\infty }y^{p}\exp\left(-{\left(\frac{y-a}{b}\right)}^{2}-x_{i}y^{2}\right)dy=e^{-\frac{a^{2}x_{i}}{1+x_{i}b^{2}}}\int_{0}^{\infty }y^{p}\exp\left(-{\left(\frac{y-\frac{a}{1+x_{i}b^{2}}}{\frac{b}{\sqrt{1+x_{i}b^{2}}}}\right)}^{2}\right)dy=e^{-\frac{a^{2}x_{i}}{1+x_{i}b^{2}}}{\left(-\frac{a}{2(1+x_{i}b^{2})}\right)}^{p+1}\Gamma (p+1)G_{1,2}^{2,0}\left[\frac{a^{2}}{b^{2}(1+x_{i}b^{2})}\;|\begin{array}{c} 1/2\\ -\frac{p+1}{2},-\frac{p}{2} \end{array}\right]$$

Now, in order to obtain simpler expressions for (48), a Corollary of Theorem 1 can be stated:

Corollary 1 *Besides the representation in terms of the H-function and of the Meijer-G function, the integral in* (34) *can also be represented in terms of the Kummer's Confluent Hypergeometric function,* F11*, as:*

(49) $$\int_{0}^{\infty }y^{\alpha_{1}}\exp\left(-{\left(\frac{y-\alpha_{2}}{\alpha_{3}}\right)}^{2}\right)dy=\frac{\alpha_{2}\alpha_{3}^{\alpha_{1}}\Gamma (\alpha_{1}+1)\sqrt{\pi }}{2^{\alpha_{1}+1}}(\frac{\alpha_{3}}{\alpha_{2}\Gamma \left(1+\frac{\alpha_{1}}{2}\right)}{}_{1}F_{1}\left[-\frac{\alpha_{1}}{2};\frac{1}{2};-\frac{\alpha_{2}^{2}}{\alpha_{3}^{2}}\right]+\frac{2}{\Gamma \left(\frac{1+\alpha_{1}}{2}\right)}{}_{1}F_{1}\left[\frac{1-\alpha_{1}}{2};\frac{3}{2};-\frac{\alpha_{2}^{2}}{\alpha_{3}^{2}}\right])$$

Proof The proof of Corollary 1 is a direct consequence of the application of the residue theorem [13] to (41). The residues of the Gamma functions inside the contour integral are simple, therefore such integral can be evaluated by the sum of two infinite series, which are found to be F11 functions. Analytical continuation allows one to write the complementary series when the argument of the Meijer-G function in (41) is greater than 1. The only tricky part of the proof is to consider that the argument of the Meijer-G function is ${\left(-a/b\right)}^{2}$ and not directly $a^{2}/b^{2}$. This is crucial in the function compositions ${\left({\left(-a/b\right)}^{2}\right)}^{(-p-1)/2}$ and ${\left({\left(-a/b\right)}^{2}\right)}^{(-p)/2}$, which show up. For simplicity, such proof is not fully presented in the present paper. An alternative proof may be obtained by using equation (1) of [17, p.230] □

This way, instead of using the Meijer-G function representation in (48), such integral can be rewritten in terms of the Confluent Hypergeometric function by means of Corollary 1, leading to:

(50) $$e^{-\frac{a^{2}x_{i}}{1+x_{i}b^{2}}}\int_{0}^{\infty }y^{p}\exp\left(-{\left(\frac{y-\frac{a}{1+x_{i}b^{2}}}{\frac{b}{\sqrt{1+x_{i}b^{2}}}}\right)}^{2}\right)dy=e^{-\frac{a^{2}x_{i}}{1+x_{i}b^{2}}}\frac{ab^{p}\Gamma (p+1)\sqrt{\pi }}{2^{p+1}{\left(1+x_{i}b^{2}\right)}^{1+p/2}}(\frac{b\sqrt{1+x_{i}b^{2}}}{a\Gamma \left(1+\frac{p}{2}\right)}{}_{1}F_{1}\left[-\frac{p}{2};\frac{1}{2};-\frac{a^{2}}{b^{2}(1+x_{i}b^{2})}\right]+\frac{2}{\Gamma \left(\frac{1+p}{2}\right)}{}_{1}F_{1}\left[\frac{1-p}{2};\frac{3}{2};-\frac{a^{2}}{b^{2}(1+x_{i}b^{2})}\right])$$

We can now focus on some particular cases of (50). At first, one should consider the following well-known formulas for the Kummer's Confluent hypergeometric function [18]:

(51) F11[a−1;b;z]=1F1[a;b;z]−zb1F1[a;b+1;z]

(52) F11[a−1;b;z]=b−2a−zb−a1F1[a;b;z]+ab−a1F1[a+1;b;z]

(53) F11[b;b;z]=exp⁡[z]

(54) F11[0;b;z]=1

(55) erf(x)=2xπF11[12,32,−x2]

in which erf(x), x∈R, is the error function.

Thus, by considering the first Kummer's Confluent hypergeometric function in (50) and using (51), one may get:

(56) F11[−p2;12;−z2]=1F1[1−p2;12;−z2]+2z21F1[1−p2;32;−z2]

As a particular case, when p=1, by using (53) and (55), the right hand side of (56) simplifies to:

(57) F11[−12;12;−z2]=e−z2+zπerf(z)

On the other hand, for p=2, by using (54), the right hand side of (56) simplifies to:

(58) F11[−1;12;−z2]=1+2z2

Now, let one consider the second Kummer's Confluent hypergeometric function in (50) and using (52), one may get:

(59) F11[1−p2;32;−z2]=2p−3+2z2p1F1[3−p2;32;−z2]+3−pp1F1[5−p2;32;−z2]

Again, when p=1, by using (54), the right hand side of (59) simplifies to:

(60) F11[0;32;−z2]=1

Also, for p=2, by using (53) and (55), the right hand side of (59) simplifies to:

(61) F11[−12;32;−z2]=(1+2z2)π4zerf(z)+e−z22

Now, by combining (47), (50), (57) and (60), $I_{1}(a,b)$ can be given explicitly as:

(62) $$I_{1}(a,b)=\sum_{i=1}^{\infty }\frac{ab\sqrt{\pi }q_{i}e^{-\frac{a^{2}x_{i}}{1+x_{i}b^{2}}}}{2{\left(1+x_{i}b^{2}\right)}^{3/2}}\left(\frac{b\sqrt{1+x_{i}b^{2}}e^{-\frac{a^{2}}{b^{2}(1+x_{i}b^{2})}}}{a\sqrt{\pi }}+\mathrm{erf}\left(\frac{a}{b\sqrt{1+x_{i}b^{2}}}\right)+1\right)$$

By following a similar procedure, the combination of (47), (50), (58) and (61), allows one to obtain $I_{2}(a,b)$ explicitly as:

(63) $$I_{2}(a,b)=\sum_{i=1}^{\infty }\frac{b^{3}\sqrt{\pi }q_{i}e^{-\frac{a^{2}x_{i}}{1+x_{i}b^{2}}}}{4{\left(1+x_{i}b^{2}\right)}^{3/2}}(\frac{2ae^{-\frac{a^{2}}{b^{2}(1+x_{i}b^{2})}}}{\sqrt{\pi }b\sqrt{1+x_{i}b^{2}}}+\left(1+\frac{2a^{2}}{b^{2}(1+x_{i}b^{2})}\right)\left(1+\mathrm{erf}\left(\frac{a}{b\sqrt{1+x_{i}b^{2}}}\right)\right))$$

This way, by combining (29), (31), (47) and (62), one shall get that:

(64) $$\varepsilon^{\prime }=\varepsilon_{\infty }+(\varepsilon_{DC}-\varepsilon_{\infty })\sum_{k=1}^{3}\frac{r_{k}\tau_{c,k}\sqrt{\pi }q_{i}}{\sqrt{2}T_{s,k}c_{k}}\sum_{i=1}^{\infty }\frac{e^{-\frac{w^{2}\tau_{c,k}^{2}x_{i}}{1+2x_{i}w^{2}T_{s,k}^{2}}}}{{\left(1+2x_{i}w^{2}T_{s,k}^{2}\right)}^{3/2}}(1+\frac{\sqrt{2}T_{s,k}\sqrt{1+2x_{i}w^{2}T_{s,k}^{2}}e^{-\frac{\tau_{c,k}^{2}}{2T_{s,k}^{2}(1+2x_{i}w^{2}T_{s,k}^{2})}}}{\tau_{c,k}\sqrt{\pi }}+\mathrm{erf}\left(\frac{\tau_{c,k}}{\sqrt{2}T_{s,k}\sqrt{1+2x_{i}w^{2}T_{s,k}^{2}}}\right))$$

One may also combine (30), (31), (47) and (63) to get:

(65) $$\varepsilon^{\prime \prime }=(\varepsilon_{DC}-\varepsilon_{\infty })\sum_{k=1}^{3}\frac{r_{k}wT_{s,k}\sqrt{\pi }}{c_{k}\sqrt{2}}\sum_{i=1}^{\infty }\frac{q_{i}e^{-\frac{w^{2}\tau_{c,k}^{2}x_{i}}{1+2x_{i}w^{2}T_{s,k}^{2}}}}{{\left(1+2x_{i}w^{2}T_{s,k}^{2}\right)}^{3/2}}\times (\frac{\sqrt{2}\tau_{c,k}e^{-\frac{\tau_{c,k}^{2}}{2T_{s,k}^{2}(1+2x_{i}w^{2}T_{s,k}^{2})}}}{\sqrt{\pi }T_{s,k}\sqrt{1+2x_{i}w^{2}T_{s,k}^{2}}}+\left(1+\frac{\tau_{c,k}^{2}}{T_{s,k}^{2}(1+2x_{i}w^{2}T_{s,k}^{2})}\right)\left(1+\mathrm{erf}\left(\frac{\tau_{c,k}}{\sqrt{2}T_{s,k}\sqrt{1+2x_{i}w^{2}T_{s,k}^{2}}}\right)\right))+\frac{\sigma_{DC}}{w\varepsilon_{0}}$$

Even though (64) and (65) are exact up to any given accuracy, it may be necessary more than 50 terms of the summation to get 4-digit accuracy. We can, on the other hand, take advantage of (43) and (45) to notice that ${\left(1+y^{2}\right)}^{-1}$ can be expanded in terms of exponential functions. Thus, instead of directly using the series expansion, we can propose a nonlinear approximation based on curve fitting techniques.

### 5.2 Approximate expressions

The integral $I_{p}(a,b)$ is difficult to analytically evaluate due to the function $y^{p}{\left(1+y^{2}\right)}^{-1}$. In special, for our permittivity studies, we are worried about $y{\left(1+y^{2}\right)}^{-1}$ and $y^{2}{\left(1+y^{2}\right)}^{-1}$.

Consider the function $y^{2}{\left(1+y^{2}\right)}^{-1}$. Based on the fact that ${\left(1+y^{2}\right)}^{-1}$ can be expanded in terms of exponential functions and noticing that $y^{2}{\left(1+y^{2}\right)}^{-1}=1-{\left(1+y^{2}\right)}^{-1}$ we propose the following approximation for $y^{2}{\left(1+y^{2}\right)}^{-1}$:

(66) $$\frac{y^{2}}{1+y^{2}}\approx \sum_{l=1}^{M}\lambda_{l}\left(1-\exp\left(-\beta_{l}y^{2}\right)\right)$$

By performing a nonlinear fitting procedure using the software Mathematica, it was found that M=3 is enough to obtain a maximum absolute error of about 0.0185 (around y=0.546) for the entire domain. For M=3, $\lambda_{1}=0.58974$, $\beta_{1}=1.28188$, $\lambda_{2}=0.313759$, $\beta_{2}=0.253207$, $\lambda_{3}=0.0870197$, $\beta_{3}=0.0438525$. Thus, by combining (30) and (66), one can get:

(67) $$\varepsilon^{\prime \prime }=(\varepsilon_{DC}-\varepsilon_{\infty })\sum_{k=1}^{3}\frac{r_{k}}{w^{2}T_{s,k}^{2}c_{k}}\times \sum_{l=1}^{3}\lambda_{l}\int_{0}^{\infty }\left(1-\exp\left(-\beta_{l}y^{2}\right)\right)\exp\left(-\frac{{\left(y-w\tau_{c,k}\right)}^{2}}{2w^{2}T_{s,k}^{2}}\right)dy+\frac{\sigma_{DC}}{w\varepsilon_{0}}$$

When p=0 in (50), one easily obtains:

(68) $$e^{-\frac{a^{2}x_{i}}{1+x_{i}b^{2}}}\int_{0}^{\infty }\exp\left(-{\left(\frac{y-\frac{a}{1+x_{i}b^{2}}}{\frac{b}{\sqrt{1+x_{i}b^{2}}}}\right)}^{2}\right)dy=\frac{e^{-\frac{a^{2}x_{i}}{1+x_{i}b^{2}}}b\sqrt{\pi }}{2\sqrt{1+x_{i}b^{2}}}\left(1+\mathrm{erf}\left(\frac{a}{b\sqrt{1+x_{i}b^{2}}}\right)\right)$$

This way, by using (68) with $x_{i}=0$ as well as with $x_{i}=\beta_{l}$, (67) reduces to:

(69) $$\varepsilon^{\prime \prime }=(\varepsilon_{DC}-\varepsilon_{\infty })\sum_{k=1}^{3}\frac{r_{k}}{wT_{s,k}c_{k}}\sum_{l=1}^{3}\frac{\lambda_{l}\sqrt{\pi }}{\sqrt{2}}(1+\mathrm{erf}\left(\frac{\tau_{c,k}}{\sqrt{2}T_{s,k}}\right)-\frac{e^{-\frac{w^{2}\tau_{c,k}^{2}\beta_{l}}{1+2\beta_{l}w^{2}T_{s,k}^{2}}}}{\sqrt{1+2\beta_{l}w^{2}T_{s,k}^{2}}}\left(1+\mathrm{erf}\left(\frac{\tau_{c,k}}{\sqrt{2}T_{s,k}\sqrt{1+2\beta_{l}w^{2}T_{s,k}^{2}}}\right)\right))+\frac{\sigma_{DC}}{w\varepsilon_{0}}$$

A similar procedure could be carried out to obtain an approximation to the real part of complex permittivity, but numerical experiments revealed the errors were not within a reasonable range. Therefore, only the approximation for the imaginary part of the complex permittivity is presented.

In order to illustrate the usage of the new formulas derived, we shall fit experimental blood dielectric data from the literature.

## 6 Fitting experimental data with the new formulas

**Figure 1:**
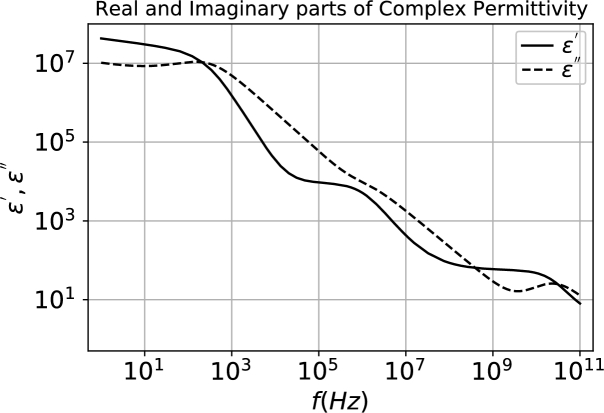
Experimental data collected by [2] for whole human blood (hematocrit value of 0.39) at 310 K.

In [2], the dielectric properties of human blood were studied using broadband dielectric spectroscopy. Those authors covered a frequency range from 1 Hz to 40 GHz, providing information on all the typical dispersion regions of biological matter. Among the published values, we chose the ones with hematocrit values of 0.39, corresponding to the whole blood samples used in [2]. Besides, we chose the samples with temperature of 310 K, which are around human body's regular temperature. Fig. 1 presents these results.

After analyzing the dataset on Fig. 1, it was indicated in [2] that there was no evidence for a low-frequency relaxation (*α*-relaxation) as well as for relaxation between *β*- and *γ*-relaxations (*δ*-relaxation).

Their analysis, on the other hand, indicated a strong Maxwell–Wagner relaxation arising from the polarization of the cell membranes in the 1–100 MHz region (*β*-relaxation) as well as an important relaxation in the microwave region beyond 1 GHz (*γ*-relaxation).

Therefore, in a similar manner as [11], we shall consider the relaxation modeling on the *β*-relaxation region. The *β*-relaxation is directly linked to RBCs [2]. Also, as also pointed out by [11], RBCs constitute almost 99 % of the blood. This allows us to consider $r_{RBC}\approx 1$ and $r_{WBC}=r_{MLP}\approx 0$

We shall use (27) and its exact and approximate version to perform the fittings. For comparison, we will use (8) of [11], which is:

(70) $$\varepsilon^{\prime \prime }=(\varepsilon_{DC}-\varepsilon_{\infty })\sum_{k=1}^{3}\int_{0}^{\infty }\frac{w\tau^{2}}{\sqrt{2\pi T_{s,k}^{2}}(1+w^{2}\tau^{2})}\exp\left(-\frac{{\left(\tau -\tau_{c,k}\right)}^{2}}{2T_{s,k}^{2}}\right)d\tau +\frac{\sigma_{DC}}{w\varepsilon_{0}}$$

**Figure 2:**
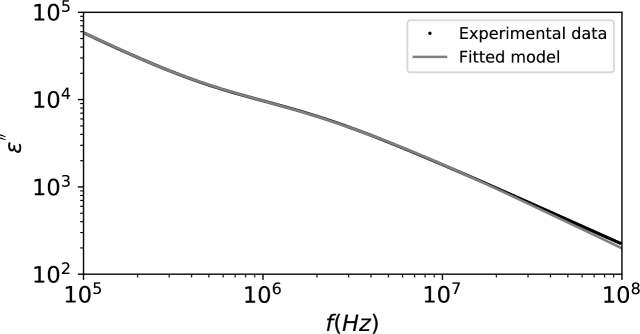
Fitted values for complex permittivity.

**Figure 3:**
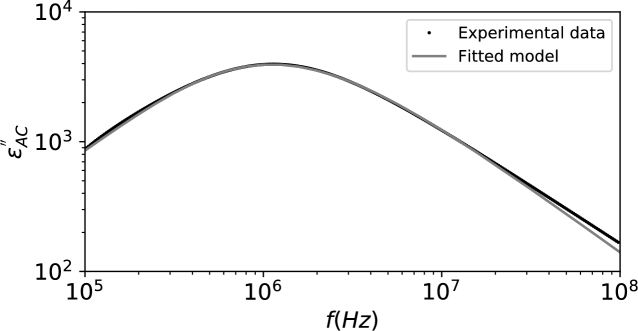
Fitted values for AC complex permittivity.

By noticing that $\varepsilon_{0}=8.854\times 10^{-12}F/m$ and using (27), the following fitting parameters were obtained: $(\varepsilon_{DC}-\varepsilon_{\infty })=8.993\times 10^{3}$, $T_{RBC}=1.139\times 10^{-7}s$, $\tau_{RBC}=2.434\times 10^{-08}s$ and $\sigma_{DC}=3.204\times 10^{-1}\Omega^{-1}m^{-1}$. On the other hand, by using (70), the fitting parameters were $(\varepsilon_{DC}-\varepsilon_{\infty })=1.533\times 10^{11}$, $T_{RBC}=1.139\times 10^{-7}s$, $\tau_{RBC}=2.434\times 10^{-08}s$ and $\sigma_{DC}=3.204\times 10^{-1}\Omega^{-1}m^{-1}$. The fittings are presented in Figs. 2 and 3. It is considered that $\varepsilon_{AC}^{\prime \prime }=\varepsilon^{\prime \prime }-\sigma_{DC}/(w\varepsilon_{0})$.

In [2], the fitted values for a Cole-Cole model were $(\varepsilon_{DC}-\varepsilon_{\infty })=9.23\times 10^{3}$ and $\sigma_{DC}=3.2\times 10^{-1}\Omega^{-1}m^{-1}$, really close to the values predicted by using (27).

As expected, while comparing the results from (27) and (70), apart from the fitted value of $\left(\varepsilon_{DC}-\varepsilon_{\infty }\right)$, all the other parameters are equal. To explain such difference, a direct comparison between (27) and (70) indicates that, in order to maintain consistency:

(71) $${\left(\varepsilon_{DC}-\varepsilon_{\infty }\right)}_{\text{[11]}}=\frac{\sqrt{2\pi }}{T_{s,k}c_{k}}r_{k}{\left(\varepsilon_{DC}-\varepsilon_{\infty }\right)}_{new}$$

where the subindex [11] indicates the value of $\left(\varepsilon_{DC}-\varepsilon_{\infty }\right)$ obtained by using the formula presented in [11] and presented in (70), while the subindex *new* is the value obtained by using (27).

It can be seen that if the results presented by [11] were used, we would obtain values of $\left(\varepsilon_{DC}-\varepsilon_{\infty }\right)$ which depended on the relative number of each cell/particle with respect to the total number of dipoles, $r_{k}$. This is not correct, as the value of $\left(\varepsilon_{DC}-\varepsilon_{\infty }\right)$ is unique for each relaxation region for the blood sample. This issue, on the other hand, was not observed in the experimental dataset we used, since only RBCs were considered and $r_{RBC}\approx 1$; $r_{WBC}=r_{MLP}\approx 0$.

Besides, it can be seen that the misuse of the Gaussian distribution also impacts the value of $\left(\varepsilon_{DC}-\varepsilon_{\infty }\right)$. A correction factor equal to $\frac{\sqrt{2\pi }}{T_{s,k}c_{k}}$ must be applied to the results in [11] to express the correct values of the parameters.

Another interesting characteristic is that the value of *τ* does not meet the peak $e_{AC}^{\prime \prime }$ value anymore. This comes from the fact that the Modified-Weibull distribution is asymmetric with respect to the mean.

It has been shown that previous results published in [11] may overestimate the value of $\left(\varepsilon_{DC}-\varepsilon_{\infty }\right)$. In the next section, the identifiability of mixtures of Modified Weibull Distributions is briefly discussed. This can be an issue while performing the fitting procedures hereby proposed.

## 7 Identifiability of mixtures of modified Weibull distributions

Prior to studying identifiability, we may first define what mixture of distributions are. In general, one may understand the mixture of distributions as a way to build multimodal distributions by linearly combining probability density functions.

Identifiability, on the other hand, can be understood as a property of a given random variable which indicates its estimated parameters are unique. This is a fundamental property to properly model dielectric relaxation, as the physical parameters estimated from experimental results will be unique and comparable.

The identifiability of some mixture models has been investigated by several authors [19], [20], [21]. In [20] it was shown that the finite mixture models with distributions: Poisson, the product of *n* exponential, Gaussian *n*-dimensional distributions and combinations of these last two distributions, Cauchy distributions, negative nondegenerate binomial distribution and families of distributions with a single location parameter are identifiable. Also, the authors in [22] proved the identifiability of finite mixture model of Weibull distributions with *m* components. Therefore, using variations of these distributions to model the relaxation times of each of the constituent particles of blood may be the first safe alternative. The identifiability of Modified Weibull distributions, as described in the present paper, is still an open question. Therefore, the results in [11] and the equations presented in the present paper must be used with caution.

The estimation of parameters from a non-identifiable distribution need extra constraints to make sure the results are comparable. For example, physically defined ranges for the parameters as well as similarity between previously published results shall be taken into account to narrow down the possible values of the fitted parameters. This issue, on the other hand, shall be the object of another paper.

## 8 Conclusions

Noninvasive tests using human blood have proven to be useful to diagnose diseases like diabetes and leukemia. In special, literature reveals that the dielectric parameters of blood are relevant for various other medical applications like cell separation, checking the deterioration of preserved blood and dielectric coagulometry.

In the present paper, we studied the statistical modeling of dielectric relaxation data for human blood, which is reported to show a deviation from the classical Debye model. We discussed and corrected some previously published results. Most of the issues were related to the statistical modeling of the dielectric characterization of human blood.

It was shown that previous results in [11] were related to Modified Weibull distributions of relaxation times, not Gaussian ones. By correcting this misconception, we could incorporate the effect of a normalizing constant which affects some fitting parameters of the model. This way, we hope to enhance the mathematical models used to fit experimental results, leading to more robust feature estimations and, ultimately, a better definition of the parameters' thresholds to classify the dielectric behavior as normal (healthy) or anomalous (sick).

Besides, novel exact and closed-form expressions for the real and imaginary parts of complex permittivities were obtained in terms of generalized hypergeometric functions. These results expand previously published relations. Also, a high accuracy approximation was created for the imaginary part of the complex permittivity when the distribution of relaxation times follow a Modified Weibull distribution.

We use the new equations to fit previously published experimental data for human blood. They showed good agreement with literature fitting results, which reinforces their validity. Finally, the identifiability of the Modified Weibull distribution has been briefly discussed, indicating this is still an open problem which shall be addressed in subsequent papers. By addressing the indicated issues, the present paper enhanced previously published models, and contributed to building more robust and reproducible test analysis methodologies.

## Declarations

### Author contribution statement

Luan de Sena Monteiro Ozelim, Charandeep Singh Sodhi, Pushpa Narayan Rathie: Analyzed and interpreted the data; Wrote the paper.

### Funding statement

This research did not receive any specific grant from funding agencies in the public, commercial, or not-for-profit sectors.

### Data availability statement

Data will be made available on request.

### Declaration of interests statement

The authors declare no conflict of interest.

### Additional information

No additional information is available for this paper.

## Appendix A

In order to better familiarize the reader, we shall present some of the special functions and mathematical relations which are used throughout the paper. We shall start with more general functions and then present some special cases.

The H-function (see [13]) is defined as a contour complex integral which contains gamma functions in their integrands, by

(72) $$H_{p,q}^{m,n}\left[z\;|\begin{array}{cccccc} (a_{1},A),\; & \ldots ,\; & (a_{n},A_{n}),\; & (a_{n+1},A_{n+1}),\; & \ldots ,\; & \left(a_{p},A_{p}\right)\\ (b_{1},B_{1}),\; & \ldots ,\; & (b_{m},B_{m}),\; & (b_{m+1},B_{m+1}),\; & \ldots ,\; & \left(b_{q},B_{q}\right) \end{array}\right]=\frac{1}{2\pi i}\int_{L}\frac{\prod_{j=1}^{m}\Gamma (b_{j}+B_{j}s)\prod_{j=1}^{n}\Gamma (1-a_{j}-A_{j}s)}{\prod_{j=m+1}^{q}\Gamma (1-b_{j}-B_{j}s)\prod_{j=n+1}^{p}\Gamma (a_{j}+A_{j}s)}z^{-s}ds,$$

where $i=\sqrt{-1}$, $A_{j}$ and $B_{j}$ are assumed to be positive quantities and all the $a_{j}$ and $b_{j}$ may be complex. The contour *L* runs from c−i∞ to c+i∞ such that the poles of $\Gamma (b_{j}+B_{j}s)$, j=1,…,m lie to the left of *L* and the poles of $\Gamma (1-a_{j}-A_{j}s)$, j=1,…,n lie to the right of *L*.

The previous representation is related to the Mellin transform of the H-function as, for any given function f(x), its direct and inverse Mellin transforms can be given as (see [23], pg. 96):

(73) $$M_{s}(f(x))=\int_{0}^{\infty }x^{s-1}f(x)dx,\;\;\text{where}\;\;f(x)=\frac{1}{2\pi i}\int_{L}x^{-s}M_{s}(f(x))ds.$$

By considering the definition in (72), the H-function can be expressed in computable form as infinite series [13]. The simpler series arise when the poles of the gamma functions in the numerator of the quotients are simple. When this simplification does not hold, residue theorem has to be applied. For details about this theorem, one may refer to [23].

Another hypergeometric function which is of interest in the present paper is the Meijer-G function, which can be obtained as a particular case of the H-function when $A_{j},B_{j}=1$, ∀*j* [13].

Also, a particular case of both the H-function and of the Meijer-G function is the Kummer's Confluente hypergeometric function, F11, defined as [13]:

(74) F11[a;b;z]=∑n=0∞(a)n(b)nznn!

where the symbols follow the same constraints as in the case of the H-function. Also, ${\left(a\right)}_{n}$ denotes the Pochhammer symbol, which can be defined in terms of the Gamma function as:

(75) $${\left(a\right)}_{n}=\frac{\Gamma (a+n)}{\Gamma (a)}$$

Both the H-function and the Meijer-G function can be related to the F11 function by the following formula:

(76) F11[a;b;z]=Γ(b)Γ(a)H1,21,1[−z|(1−a,1)(0,1),(1−b,1)]=Γ(b)Γ(a)G1,21,1[−z|1−a0,1−b]
